# *Latilactobacillus sakei* WIKIM31 Decelerates Weight Gain in High-Fat Diet-Induced Obese Mice by Modulating Lipid Metabolism and Suppressing Inflammation

**DOI:** 10.4014/jmb.2107.07024

**Published:** 2021-09-15

**Authors:** Sung-Soo Park, Seul Ki Lim, Jieun Lee, Hyo Kyeong Park, Min-Sung Kwon, Misun Yun, Namhee Kim, Young Joon Oh, Hak-Jong Choi

**Affiliations:** 1 Microbiology and Functionality Research Group, World Institute of Kimchi, Gwangju 61755, Republic of Korea; 2 SME Service Department, Strategy and Planning Division, Gwangju 61755, Republic of Korea

**Keywords:** *Latilactobacillus sakei* WIKIM31, probiotics, obesity, inflammation

## Abstract

Obesity and related metabolic diseases are major problems worldwide. Some probiotics are currently considered potential therapeutic strategies for obesity. We aimed to investigate the antiobesity efficacy of *Latilactobacillus sakei* WIKIM31 in obese mice induced by a high fat diet. The administration of a high-fat diet with *L. sakei* WIKIM31 reduced body weight gain, epididymal fat mass, triglyceride and total cholesterol levels in the blood, and remarkably decreased the expression of lipogenesis-related genes in the epididymal adipose tissue and liver. Interestingly, intake of *L. sakei* WIKIM31 improved gut barrier function by increasing the gene expression of tight junction proteins and suppressing the inflammatory responses. Additionally, *L. sakei* WIKIM31 enhanced the production of short-chain fatty acids, such as butyrate and propionate, in the intestinal tract. These results showed that *L. sakei* WIKIM31 can be used as a potential therapeutic probiotic for obesity.

## Introduction

Obesity is a serious disease worldwide and is associated with many metabolic diseases, such as diabetes, fatty liver, and cardiovascular diseases [1]. Recent studies have shown that the gut microbiome, which manifested by changes in bacterial compositions and function, is closely related to obesity [2]. Changes in specific intestinal microbiota, particularly, the ratio of the phyla *Firmicutes* and *Bacteroidetes*, which are representative dominant bacteria, is the main cause of obesity [3]. These changes in dominant bacteria affect the energy metabolism of the host, which can cause metabolic problems [4]. In addition, emerging research indicates that chronic inflammation is highly associated with the development of obesity [5]. Thus, obese individuals have increased circulating levels of interleukin (IL)-6, tumor necrosis factor (TNF)-α, and monocyte chemoattractant protein-1 (MCP-1) [6]. Obesity induced inflammation is related with leakage of microbial, gut-derived molecules, such as lipopolysaccharide (LPS) [7]. Alterations in gut microbiota induced by a high-fat diet cause intestinal inflammation, which leads to the leakage of gut microbes and contributes to inflammation associated with obesity [8]. Therefore, modulation of the gut microbiota is very important as a potential therapeutic strategy for metabolic disorders.

Probiotics, as living bacteria, not only contribute to the health of the gastrointestinal tract, but also have a positive effect on health when administered in appropriate amounts to the host [9]. Other beneficial effects of probiotics include improving blood glucose, cholesterol, hypertension, immune response, and energy metabolism [10]. Several studies have shown that probiotic bacteria inhibit the lipid metabolism and differentiation of adipocytes [11, 12]. Although a number of studies have been reported on the beneficial effects of probiotics on host metabolism, the mechanisms by which probiotics modulate host metabolism remain to be elucidated.

*L. sakei* WIKIM31 was isolated from kimchi. Kimchi is fermented by various lactic acid bacteria, of which *L. sakei* is one of the representative dominant bacteria [13]. Previous studies have shown that *L. sakei* inhibits the growth of harmful bacteria and has the immunomodulatory effects by inhibiting of allergic Th2 responses and increasing the production anti-inflammatory cytokines [14, 15]. Also, *L. sakei* has anti-adipogenesis and anti-obesity effects [16, 17]. In this study, we investigated whether oral administration of WIKIM31 could suppress lipid accumulation in adipocytes by regulating the expression of lipogenesis genes, inflammatory cytokines, and tight junction genes in vivo.

## Materials and Methods

### Isolation and Preparation of *L. sakei* WIKIM31

*L. sakei* WIKIM31 was isolated from homemade kimchi (Gangwon province, Republic of Korea). The kimchi was homogenized and filtered by stomacher filter bag. The homogenate was spread onto Man-Rogosa-Sharpe (MRS; BD Difco, USA) agar. The plate were incubated to at 30°C for 48 h. Isolated strains were obtained from subculture. After pre-screening for anti-obesity effects in vitro, selected strain was identified by 16S rRNA gene sequence. WIKIM31 was deposited at Korean Federation of Culture Collection as KFCC 11654P. To prepare the strain for use in animal experiments. WIKIM31 was cultured at 30°C for 18 h in MRS broth. The strain was harvested by centrifugation and washed twice with PBS. Next, the strain was resuspended in PBS (1 × 10^9^ CFU) for oral administration to mice.

### Cell Culture and Oil Red O Staining

3T3-L1 (ATCC-CL-173) preadipocyte cells were maintained in Dulbecco Modified Eagle Medium (DMEM; Gibco, USA) supplemented with 10% newborn calf serum (Gibco), 100 U/ml penicillin, and 100 μg/ml streptomycin. For differentiation to adipocytes, preadipocytes were cultured to full confluency, and the medium was changed with differentiation media (DMEM supplemented with 10% fetal bovine serum [FBS]) with 1 μg/ml insulin, 0.1 μM dexamethasone, and 0.5 mM isobutyl methylxanthine. After 2 days, the cells were cultured in DMEM containing 10% FBS for additional 4 days. For Oil Red O staining, cells were washed with PBS, fixed with 10% formalin for 1 h, and rinsed with 60 % isopropyl alcohol. Cells were stained with Oil Red O solution for 10 min, and washed with distilled water.

### Animals and Experimental Design

The care and study of the laboratory mice followed the protocols of the Institute Animal Care and Use Committee for the World Institute of Kimchi (WIKIM IACUC 201608). 6-week-old male C57BL/6 mice were purchased from OrientBio (Korea) and acclimated to the environment for 1 week. All mice were cared in individually ventilated cages where temperature and humidity were controlled on a 12 h light/dark cycle. Food and water were supplied ad libitum. Each group of mice were fed a normal diet (ND mice, 10% kcal as fat, D10001, Research Diet Inc., USA), high-fat diet (HFD mice, 45% kcal fat, D12451, Research Diet), or HFD with WIKIM31 (HFD-WIKIM31 mice) for 12 weeks. WIKIM31 was orally administered at a daily (10 am) concentration of 1 × 10^9^ CFU per 200 μl. The well-being of the animals, body weight, and food intake were measured weekly. The body composition of the mice was determined by MRI relaxometry (EchoMRI-500, Echo Medical System, USA). After 12 weeks, the mice were sacrificed using CO_2_ asphyxiation. Serum separated from blood was stored at -80°C until analysis.

### Histological Analysis

Histological analysis was performed as described previously [18]. Histological images of the liver and adipose tissue were examined under a microscope (Olympus DP73, Japan).

### Measurements of Triglyceride, Cholesterol, Glucose and Adipokines Levels

Serum triglyceride, cholesterol, and glucose levels were determined by a FUGI DRI-CHEM 7000 (Fujifilm, Japan) according to the manufacturer’s protocol. The concentrations of leptin, adiponectin, and resistin were measured using a Bio-Plex Pro Mouse Diabetes-Plex Assay (Bio-Rad, USA).

### Isolation of Stromal Vascular Fraction and Measurements of Cytokine Levels

Isolation of stromal vascular fraction (SVF) was performed as previously described [18]. Briefly, epididymal fat (EF) tissues were digested using 2 mg/ml collagenase (Sigma-Aldrich, USA) at 37°C for 45 min, filtered using 100 μm cell strainers (BD Falcon), and centrifugated at 300 ×*g* for 10 min at room temperature. The adipocyte layer and the supernatant were separated, and SVF pellets were collected. For the measurement of pro-inflammatory cytokine secretion, the isolated SVFs (5 × 10^5^ cells/well) were seeded in a 96-well plate, added to LPS (100 ng/ml), and cultured for 24 h at 37°C. Levels of IL-6, TNF-α, and MCP-1 in the cultured supernatant were measured using a cytometric bead array kit (BD Biosciences, USA).

### RNA Isolation and Real-Time PCR Analysis

Total RNA was isolated from frozen tissues and cDNA was synthesized using a TOPscript cDNA synthesis kit (Enzynomics, Korea). Real-time PCR was performed using a CFX9600 (Bio-Rad). PCR experiments were performed under the same cycling conditions: 95°C for 5 min, followed by 39 cycles of 95°C for 15 s, 55°C for 15 s, and 72°C for 15 s. The PCR primer sequences are listed in Table 1. β-Actin RNA levels were used to normalize each gene.

### Statistical Analysis

All data are presented as the mean ± standard error (S.E.). Student’s *t*-test was used for the statistical analysis of data. Analysis of variance (ANOVA) was used to determine the level of significance.

## Results

### Selection and Identification of a Candidate Strain with Anti-Obesity Efficacy

**Fig. 1:**
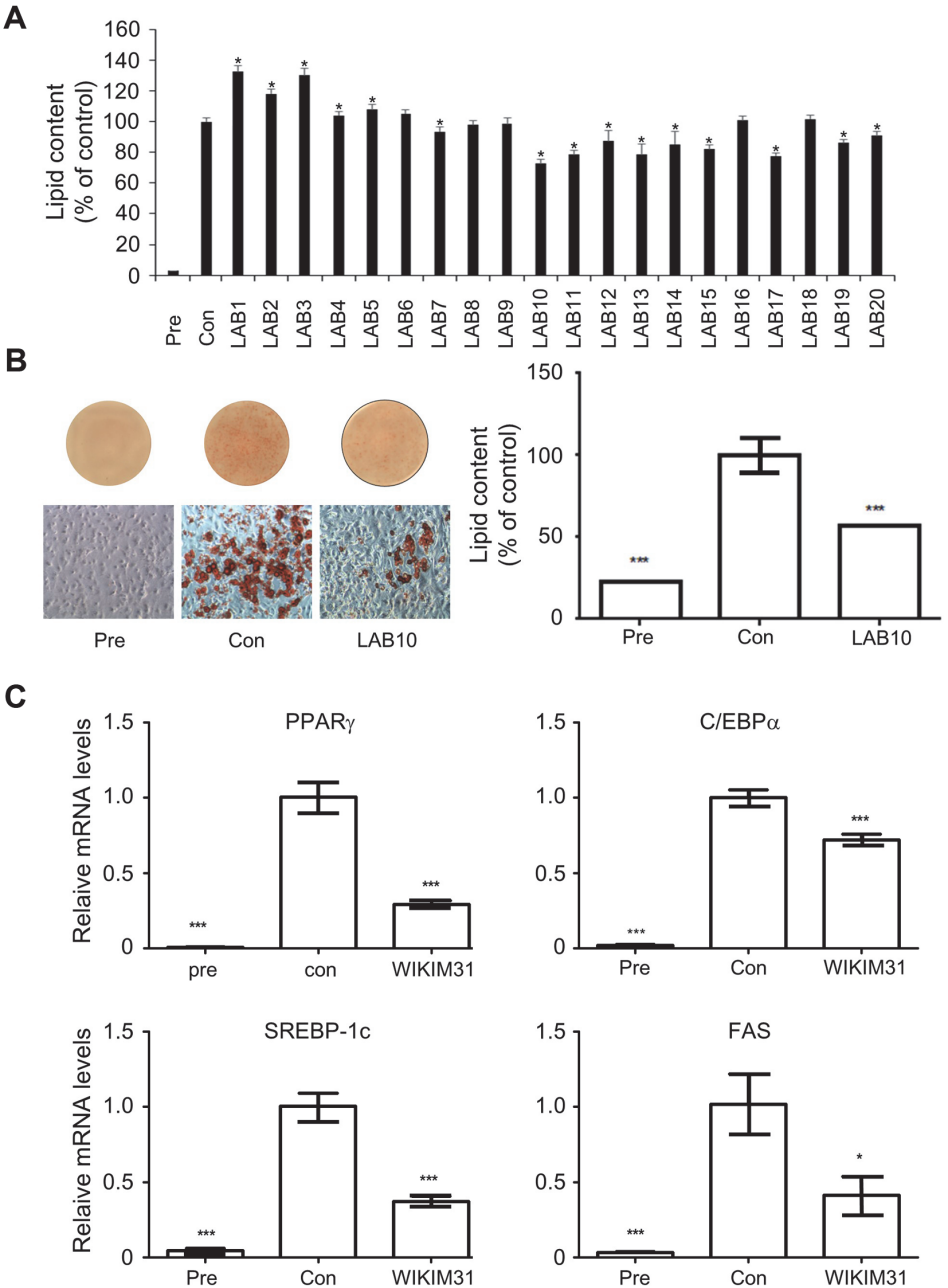
The inhibitory effect of *L. sakei* WIKIM31 on lipid accumulation. (**A, B**) 3T3-L1 cells were treated with 10% bacterial extracts for 10 days, and then intracellular lipid were stained with Oil Red O (Pre, no differentiated cells; Con, fully differentiated cells) (**C**) Gene expression of lipogenesis were determined by real-time RT-PCR. LAB10 was renamed as WIKIM31. Data are presented as the mean ± SD of triplicate tests. *p < 0.05, ***p < 0.001 vs. Con.

To screen lactic acid bacteria (LAB) with high anti-obesity efficacy in vitro, we measured the level of lipid accumulation by LAB treatment in 3T3-L1 adipocytes. Among 20 isolates, we selected a LAB10 strain for further study as it showed the highest inhibition of lipid accumulation in 3T3-L1 adipocytes (Fig. 1A). Even in a repeated assay, LAB10 reduced by about 44% compared to the control (Fig. 1B). In addition, we monitored the expression of genes related to adipogenes to assess whether WIKIM31 could suppress adipogenesis. The expression of PPARγ, C/EBPα, SREBP-1c, and FAS remarkably decreased compared with the control (Fig. 1C). Analysis of the 16S rRNA gene sequences of LAB10 showed 99.9% similarity with *Latilactobacillus sakei* (data not shown). Therefore, we identified a LAB10 strain as *L. sakei*. Based on these results, *L. sakei* LAB10 (= WIKIM31) was selected as a candidate with anti-obesity efficacy.

### *L. sakei* WIKIM31 Administration Suppresses Weight Gain in HFD-Induced Obese Mice

**Fig. 2:**
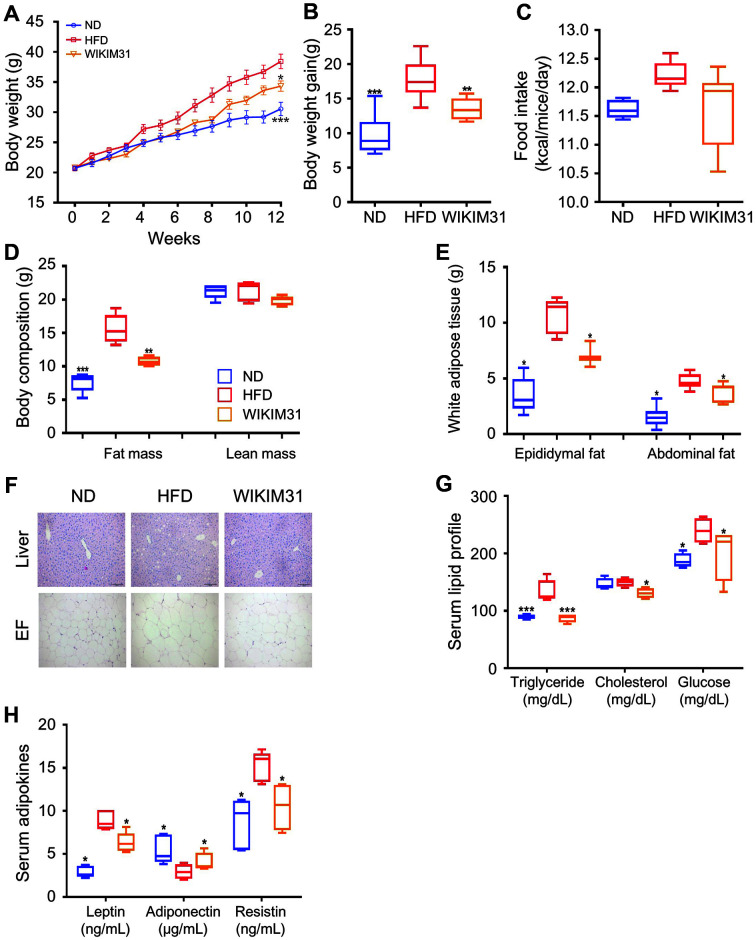
The effect of *L. sakei* WIKIM31 administration on HFD-induced obesity. WIKIM31 or PBS was administered daily to C57BL/6 mice for 12 weeks during feeding with an ND or HFD. (**A**) Body weight. (**B**) Gain in body weight. (**C**) Food intake. (**D**) Total white fat mass. (**E**) Partial white fat mass. (**F**) Representative images of liver and epididymal fat (EF) staining. (**G, H**) Serum levels of triglycerides, cholesterol, glucose, leptin, adiponectin, and resistin. Data are presented as the mean ± SE. *p < 0.05, **p < 0.01 vs. HFD.

To determine whether WIKIM31 can improve HFD-induced obesity, WIKIM31 was orally administered to 7 week old male mice for 12 weeks. Mice fed both HFD and WIKIM31 showed reduced weight gain compared to mice fed a HFD (Fig. 2A, *p* = 0.0148; B, *p* = 0.0022), although food intake was not different (Fig. 2C). WIKIM31 led to a significant reduction in fat mass gain (29%), without suppressing lean mass gain, compared to HFD mice (*p* = 0.0049) (Fig. 2D). In addition, the weights of epididymal (*p* = 0.01) and abdominal adipose tissue (*p* = 0.0113) in HFD-WIKIM31 mice were significantly lower than those in HFD mice (Fig. 2E). Consistently, adipocyte size in EF and lipid droplet deposition in the liver were reduced by WIKIM31 administration (Fig. 2F). Furthermore, the serum levels of triglycerides, cholesterol, and glucose in HFD-WIKIM31 mice were reduced by 35% (*p* = 0.0006), 13% (*p* = 0.0155), and 16% (*p* = 0.0195), respectively (Fig. 2G). Alterations in serum adipocytokine levels are closely associated with obesity. For example, serum leptin and resistin levels increased, whereas adiponectin levels decreased in HFD mice. Therefore, we investigated whether WIKIM31 administration to mice during HFD feeding affected the serum adipocytokine levels. Leptin (*p* = 0.0072) and resistin (*p* = 0.0098) serum levels decreased, and adiponectin (*p* = 0.0177) levels in HFD-WIKIM31 mice increased (Fig. 2H). Together, these results indicate that WIKIM31 administration suppresses HFD-induced obesity.

### *L. sakei* WIKIM31 Administration Alters Metabolic Gene Expression in HFD-Induced Obese Mice

**Fig. 3:**
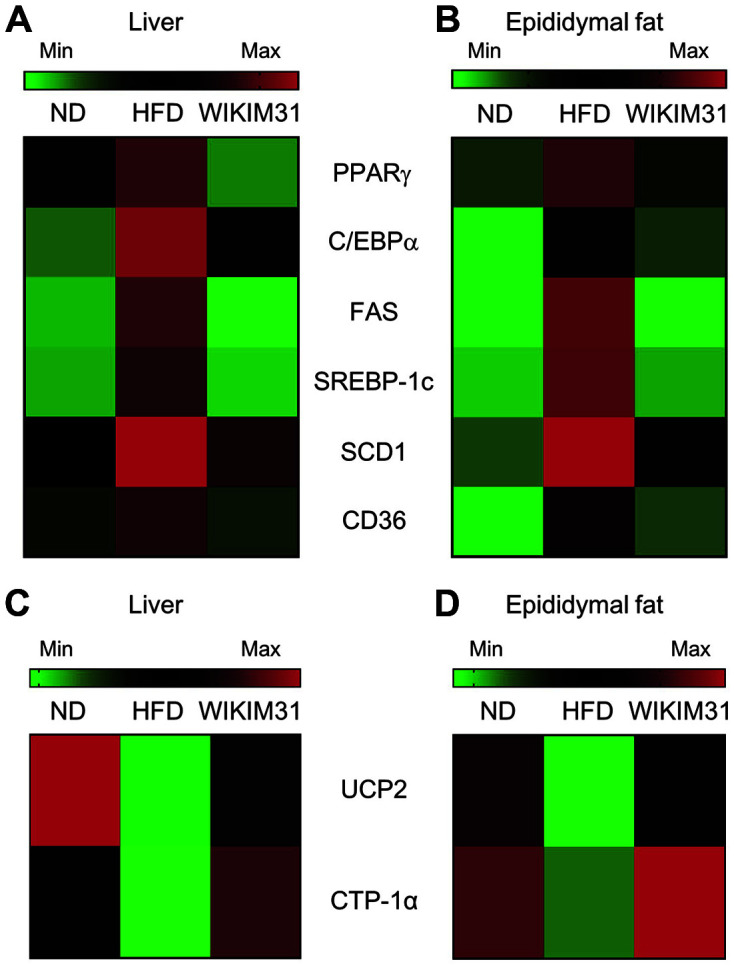
The effect of *L. sakei* WIKIM31 treatment on lipogenesis and energy metabolism. (**A, B**) The mRNA levels of lipogenesis and (**C, D**) energy metabolic genes were determined by real-time RT-PCR in liver and epididymal fat. Data are presented as the mean ± SE. *p < 0.05 vs. HFD.

To identify the mechanism underlying the phenotype of HFD-WIKIM31 mice, the expression of lipid metabolic genes in the liver and EF was determined by real-time RT-PCR. The expression levels of hepatic or EF of lipogenesis-related genes, such as PPARγ (liver, *p* = 0.023; EF, *p* = 0.0043) , C/EBPα (liver, *p* = 0.0088; EF, *p* = 0.034), FAS (liver, *p* = 0.024; EF, *p* = 0.0009), SREBP-1c (liver, *p* = 0.0042; EF, *p* = 0.0044), SCD1 (EF, *p* = 0.0456), and CD36 (liver, *p* = 0.0103; EF, *p* = 0.0117) were substantially lower in HFD-WIKIM31 mice than in HFD mice (Figs. 3A and 3B). However, the expression of UCP2 (liver, *p* = 0.0204; EF, *p* = 0.0005) and CPT-1α (liver, *p* = 0.0019; EF, *p* = 0.0001) genes involved in β-oxidation, was remarkably increased in HFD-WIKIM31 mice (Figs. 3C and 3D). These results indicate that WIKIM31 administration improves lipid and energy metabolism in the liver and adipose tissue by modulating the expression of lipogenesis -and β-oxidation-related genes.

### *L. sakei* WIKIM31 Treatment Reduces Inflammatory Response and Ameliorates Gut Barrier Function in HFD-Induced Obese Mice

**Fig. 4:**
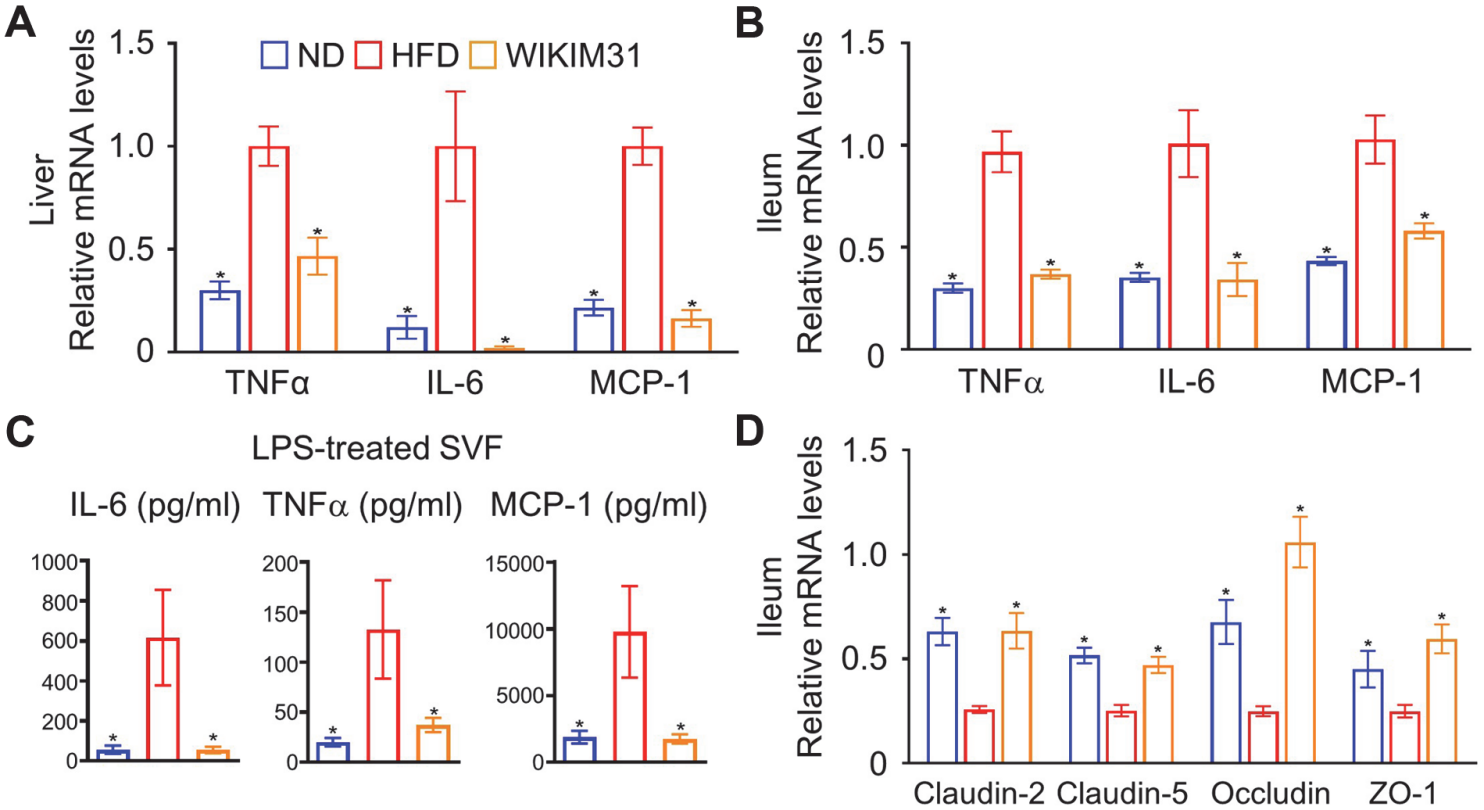
The effect of *L. sakei* WIKIM31 administration on gut barrier function and inflammation response. (**A, B**) Gene expression of pro-inflammatory cytokines was determined by real-time RT-PCR. (**C**) After isolated SVF were treated with LPS for 24 h, the levels of IL-6, TNF-α, and MCP-1 in the supernatant were detected using the Cytometric Bead Array kit. (**D**) Gene expression of tight junction proteins was determined by real-time RT-PCR. Data are presented as the mean ± SE. *p < 0.05 vs. HFD.

It is known that the secretion of pro-inflammatory cytokines and gene expression increases in HFD-induced obesity [5]. Therefore, we examined the expression levels of pro-inflammatory cytokine genes to determine whether WIKIM31 administration could reduce the inflammatory response. The expression of TNF-α, IL-6, and MCP-1, pro-inflammatory gene, were remarkably increased in the liver and ileum of HFD mice compared to those in ND mice. However, WIKIM31 administration strongly reduced the expression of these genes (TNF-α, *p* = 0.0048 in liver; *p* = 0.0003 in ileum, IL-6; liver: *p* = 0.0042, ileum: *p* = 0.0058, and MCP-1; liver: *p* = 0.0001, ileum: *p* = 0.0052) (Figs. 4A and 4B). Obesity is associated with chronic inflammation characterized by macrophages and monocytes infiltration, and pro-inflammatory cytokines production [5]. To address whether oral administration of WIKIM31 regulates the suppression of inflammatory response in the adipose tissue, SVF isolated from EF were re-stimulated with LPS for 24 h, and the levels of pro-inflammatory cytokines in the supernatant were measured. The increased levels of IL-6 (*p* = 0.047), TNF-α (*p* = 0.041), and MCP-1 (*p* = 0.0489) production in the HFD mice were remarkably decreased in the WIKIM31 mice (Fig. 4C). Intestinal inflammation is associated with intestinal barrier dysfunction [19, 20]. The effect of oral administration of WIKIM31 on the expression of tight junction proteins in the ileum of mice was further explored. Compared to HFD mice, WIKIM31 administration upregulated the expression levels of tight junction protein genes, such as claudin-2 (*p* = 0.0019), claudin-5 (*p* = 0.0017), occluding (*p* = 0.001), and zonula occludens-1 (ZO-1) (*p* = 0.0018) (Fig. 4D). Taken together, these results indicate that WIKIM31 treatment suppresses the immune responses related to obesity and further improves intestinal barrier function.

### *L. sakei* WIKIM31 Administration Increases SCFA Content in HFD-Induced Obese Mice

**Fig. 5:**
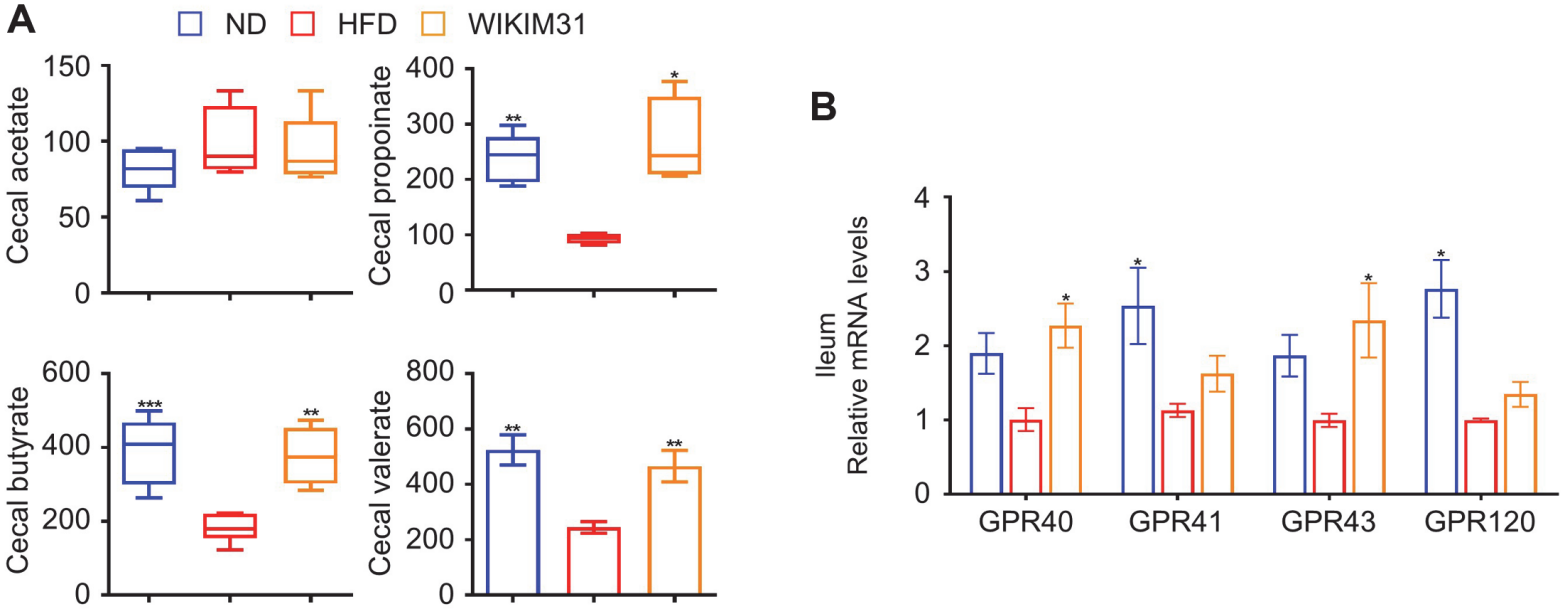
Concentration of short-chain fatty acids (SCFAs) from cecal contents and the gene expression of G protein receptors (GPRs) by *L. sakei* WIKIM31. (**A**) Content of SCFAs (total SCFAs, acetate, propionate, butyrate, and valerate) in cecum. (**B**) Gene expression of GPRs in ileum was determined by real-time RT-PCR. Data are presented as mean ± SE. *p < 0.05, **p < 0.01, ***p < 0.001 vs. HFD.

SCFAs are metabolites of the intestinal microbiota and are beneficial to the host body [21]. The contents of propionate, butyrate, and valerate in HFD mice were distinctly lower than those in ND mice (Fig. 5A). After WIKIM31 administration, these SCFAs increased in comparison with HD mice (propionate, *p* = 0.0014; butyrate, *p* = 0.0006; valerate, *p* = 0.0035). However, acetate content was not significantly different. Consistent with these results, the expression of G protein-coupled receptor (GPR) 40 (*p* = 0.0102) and GPR43 (*p* = 0.038), known as receptors for SCFAs, was remarkably increased (Fig. 5B).

## Discussion

The pharmacological approach to the treatment of obesity can cause serious side effects, and probiotic administration has been considered an alternative [22]. Several clinical trials and studies have demonstrated the usefulness of probiotics in the treatment of metabolic diseases [23]. Probiotics improve obesity status by regulating the host’s energy metabolism and gut microbiota [24, 25].

In this study, to obtain LAB with anti-obesity from kimchi, we isolated 20 LAB strains from kimchi and examined their ability to inhibit lipid accumulation in vitro. *L. sakei* WIKIM31 was selected as it showed the highest inhibitory effect. Our in vivo results showed that the oral administration of *L. sakei* WIKIM31 in HFD-induced obese mice remarkably reduced body weight and fat mass gain without reducing food intake (Fig. 2). The liver and white adipose tissues are central organs with metabolic and lipogenic functions in the body [1, 26]. Therefore, dysregulation of energy metabolism in these organs is one of potential factors for metabolic disorders, such as obesity and diabetes [27]. It has been previously reported that *L. sakei* strains OK67 and ADM14 isolated from kimchi induce anti-obesity effects by modulating lipid metabolism in adipose tissue [16, 17]. Similarly, *L. sakei* WIKIM31 administration effectively reduced serum triglyceride contents by inhibiting the expression of lipogenesis-related genes (PPARγ, C/EBPα, FAS, SREBP-1c, SCD1, and CD36) and promotes energy expenditure by increasing the expression of β-oxidation-related genes, UCP2 and CPT-1α in liver and white adipose tissues.

In obese individuals, as tight junction protein levels decrease, intestinal permeability increases, resulting in the infiltration of pro-inflammatory molecules that lead to inflammatory responses [28, 29]. Tight junction proteins such as occludin and claudins, play important roles in the intestinal barrier [30]. Previous studies have reported that ingestion of probiotic strains increases the expression of tight junction proteins in the intestine [16-18]. In the present study, *L. sakei* WIKIM31 not only increased the expression of tight junction protein genes to the level of the normal group, but also reduced the expression of pro-inflammatory cytokines in the ileum. GPRs, receptors for SCFAs, have been known to modulate the expression of tight junction proteins upon activation, as well as to improve intestinal inflammation and host energy metabolism [21, 31, 32]. However, it is unclear whether *L. sakei* can regulate the expression of GPRs in the intestine. In this study, we clearly demonstrated that *L. sakei* WIKIM31 administration increases the level of propionate and butyrate, resulting in the enhanced expression of their receptors, GPR41 and GPR43.

In conclusion, oral administration of *L. sakei* WIKIM31 ameliorated obesity by suppressing lipogenesis and inflammatory responses via improvement of gut barrier functions and the production of SCFAs. These results suggest that *L. sakei* WIKIM31 can be used as a therapeutic agent for obesity.

## Figures and Tables

**Table 1:** List of oligonucleotide primer sequences for real time RT-PCR.

Gene	Sense	Antisense
PPARγ	5′-GCCCTTTGGTGACTTTATGGA-3′	5′-GCAGCAGGTTGTCTTGGATG-3′
C/EBPα	5′-TGCTCTGATTCTTGCCAAA-3′	5′-ACCCAAAATCCCTAAACC-3′
FAS	5′-TGTTCCTTGTGCACCCCATT-3′	5′-GTAGGTGTGTGAGCCGTCAA-3′
SREBP-1c	5′-AGATCCAGGTTTGAGGTGGG-3′	5′-AGATCCAGGTTTGAGGTGGG-3′
SCD1	5′-CCTCCGGAAATGAACGAGAGAA-3′	5′-CCTGATAGGTGGGGTCGTGA-3′
CD36	5′-CTTCACAGTTCTGAATCTGGCTGT-3′	5′-GAGGCTGCGTCTGTGCCATTAATCATG-3′
UCP2	5′-AGAACGAGACACCTTTAGAGA-3′	5′-GAAGATGGAGAGAAATTGGAGAA-3′
CTP-1α	5′-ACCCTGAGGCATCTATTGACAG-3′	5′-ATGACATACTCCCACAGATGGC-3′
TNF-α	5′-ACGGCATGGATCTCAAAGAC-3′	5′-AGATAGCAAATCGGCTGACG-3′
IL-6	5′-CCTTCCTACCCCAATTTCCAA-3′	5′-AGATGAATTGGATGGTCTTGGTC-3′
MCP-1	5′-GTGACTCGGACTGTGATG-3′	5′-CATTGAAAGTGTTGAATCTG-3′
Claudin-2	5′-TATGTTGGTGCCAGCATTGT-3′	5′-TCATGCCCACCACAGATATA-3′
Claudin-5	5′-GCAAGGTGTATGAATCTGTGCT-3′	5′-GCAAGGTGTATGAATCTGTGCT-3′
Occludin	5′-TTGAAAGTCCACCTCCTTACAGA-3′	5′-CCGGATAAAAAGAGTACGCTGG-3′
ZO-1	5′-GCCGCTAAGAGCACAGCAA-3′	5′-TCCCCACTCTGAAAATGAGGA-3′
GPR40	5′-CCCACGCTAAACTGCGACT-3′	5′-CGCTGAGAGCAGCTAGGAAG-3′
GPR41	5′ -CTAAACCTGACCATTTCGGACC-3′	5′-GATAGGCCACGCTCAGAAAAC-3′
GPR43	5′-ATCCTCCTGCTTAATCTGACCC-3′	5′-CGCACACGATCTTTGGTAGG-3′
GPR120	5′-TGCCCCTCTGCATCTTGTTC-3′	5′-CGCGATGCTTTCGTGATCTG-3′
β-actin	5′-GTTACCACTGGGACGAC-3′	5′-CTCAAACATGATCTGGGTCA-3′
